# Multiple instance learning based classification of diabetic retinopathy in weakly-labeled widefield OCTA en face images

**DOI:** 10.1038/s41598-023-35713-4

**Published:** 2023-05-29

**Authors:** Philipp Matten, Julius Scherer, Thomas Schlegl, Jonas Nienhaus, Heiko Stino, Michael Niederleithner, Ursula M. Schmidt-Erfurth, Rainer A. Leitgeb, Wolfgang Drexler, Andreas Pollreisz, Tilman Schmoll

**Affiliations:** 1 Center for Medical Physics and Biomedical Engineering, Medical University of Vienna, Waehringer Guertel 18-20 (4L), 1090 Vienna, Austria; 2 Department of Ophthalmology and Optometry, Medical University of Vienna, Waehringer Guertel 18-20, 1090 Vienna, Austria; 3 Carl Zeiss Meditec Inc, 5300 Central Pkwy, Dublin, CA 94568 USA

**Keywords:** Applied physics, Diagnostic markers, Preclinical research, Scientific data

## Abstract

Diabetic retinopathy (DR), a pathologic change of the human retinal vasculature, is the leading cause of blindness in working-age adults with diabetes mellitus. Optical coherence tomography angiography (OCTA), a functional extension of optical coherence tomography, has shown potential as a tool for early diagnosis of DR through its ability to visualize the retinal vasculature in all spatial dimensions. Previously introduced deep learning-based classifiers were able to support the detection of DR in OCTA images, but require expert labeling at the pixel level, a labor-intensive and expensive process. We present a multiple instance learning-based network, MIL-ResNet,14 that is capable of detecting biomarkers in an OCTA dataset with high accuracy, without the need for annotations other than the information whether a scan is from a diabetic patient or not. The dataset we used for this study was acquired with a diagnostic ultra-widefield swept-source OCT device with a MHz A-scan rate. We were able to show that our proposed method outperforms previous state-of-the-art networks for this classification task, ResNet14 and VGG16. In addition, our network pays special attention to clinically relevant biomarkers and is robust against adversarial attacks. Therefore, we believe that it could serve as a powerful diagnostic decision support tool for clinical ophthalmic screening.

## Introduction

Diabetic retinopathy (DR) is the most common microvascular complication of working-aged adults suffering from diabetes mellitus (DM) and affects retinal functionality, potentially resulting in blindness^1^. Therefore, it poses a major healthcare challenge^2^. It was estimated that 35% of all diabetic patients suffer from any form of DR and 7% have advanced stages of DR. The severity of DR can be divided into a nonproliferative (mild, moderate, severe), and a proliferative form^3,4^. Since DR progresses asymptomatically in its early stages, i.e. the nonproliferative occurrences, the only way for a timely diagnosis are regular ophthalmological examinations^5^ and monitoring the progression over time^6,7^. DR lesions occurring predominantly in the retinal periphery and their importance in assessment of disease severity, as well as risk for progression, have received a lot of attention as of lately^8–13^. Therefore, identifying peripheral changes, requiring a large field of view (FoV), is of clinical relevance.

Multiple optical imaging techniques have been utilized to assess pathological diabetes-related changes of the retina, including fundus photography (FP)^9,14^, optical coherence tomography (OCT)^15,16^, fluorescein angiography (FA)^8,12,17^, and most recently optical coherence tomography angiography (OCTA)^18–21^. OCTA is a non-invasive functional extension of OCT^22^, capable of visualizing blood flow of the microvasculature in the retina in a non-invasive way^23,24^. Widefield OCTA provides tremendous clinical benefits in modern Swept-Source (SS)-OCT systems^25–27^. Advances in the continuous improvements of laser A-scan rates will allow for imaging of the retina’s periphery in ever-increasing FOVs. However, ultra-widefield FP still provides a much larger FOVs, which can only be matched if OCTA imaging can cover areas of the same size with little or no segmentation errors.

Recently published work on the detection and classification of DR increasingly relies on some kind of artificial intelligence (AI)-support, in particular, deep learning (DL)-algorithms^28^. Many earlier AI-assisted approaches had labeled or even semantically segmented FP-images as the source of information for the decision-making process of the classification algorithms^29–31^. Islam et al. showed in their systematic review that the detection of DR via FP worked quite accurately for most DR patients in various publications^32^. However, pure FP cannot visualize the vasculature of the retina with sufficient detail.

Our work aims to find signs of changes in the vasculature and especially the periphery of a clinical widefield OCTA data set that was acquired with the *Department of Ophthalmology* at the *Medical University of Vienna*.

Past approaches, utilizing OCT, focused on the reflectivity of retinal layers taken from segmented OCT-scans^33^ or the spatial appearance of the retina, were based on segmented 3D-OCT scans^5^. Other approaches utilized the vessel density^34^, or capillary nonperfusion, both derived from clinical OCTA data of DR patients^13^.

Widefield OCTA en face images intrinsically do not display obvious clinical signs, i.e. biomarkers at every portion of the image. However, identifying those areas that show pathological changes is crucial for the correct classification of an image. For the database utilized in this work, the only label we obtain is whether or not a patient is diabetic.

Labeling widefield OCTA images is challenging since a lot of areas are ambiguous without knowledge of the whole dataset. Missing labels in a dataset, which in our case are semantic annotations in the OCTA en face images, is a well-known issue in machine learning (ML). Almost all previously described supervised ML-approaches rely on accurately semantically segmented data and severity labels, provided in most cases by expert graders. This process is very labor-intensive, expensive, and subjective since it very much depends on the experience of the grader.

Multiple instance learning (MIL), a technique that is subordinated in the field of supervised learning (SL), constitutes an elegant method to use weakly-labeled data. Previously, researchers have used technologies such as FP to collect images as training sets and used multiple instance-based learning to detect DR^35^. In MIL, a large labeled object, called a bag, is divided into several sub-objects, so-called instances. These instances do not contain individual labels, only inherit the global label from the bag from which they were taken^36,37^. Classification usually follows one of two principles: *instance-level* algorithms and *metadata-based* or *bag-level* algorithms, with the latter being the more commonly used approach in MIL. Instance-level classification approaches learn representative instance features from a set of instance feature vectors in one bag to classify future bags. *Bag-level* classification tries to conceptualize instance-independent information from the bags themselves^36^.

With the work presented in this manuscript, we show that weakly-labeled clinical widefield OCTA en face image data holds all information for a potential early diagnosis of DR in patients suffering from DM. We, therefore, conclude that data without strong labels allows for picking up on sufficient features in OCTA en face images indicating diagnostically relevant structural changes detectable for accurate classification of DR.

## Methods

In the following section, we elaborate on how we processed and made use of the clinical widefield OCTA en face data, including pre-processing, training, and evaluation of all utilized network architectures. Since our network utilizes MIL, a supervised learning classification approach, we benchmarked our network against two other proven single instance learning (SIL)-based networks for classification, ResNet14, and VGG16^38–40^. Da Rocha et al. could show that a pre-trained version of VGG16 is capable of predicting DR from FP^41^. Since it is rather hard for a human to trace what portions of the images a convolutional neural network (CNN) deems important, we implemented a visualization technique called Grad-CAM^42^, highlighting regions contributing strongly to the classification decision, ideally clearly visible biomarkers.

### OCTA dataset and data acquisition

Our dataset was acquired with a modified PLEX^®^ Elite 9000 SS-OCT system, with an Fourier domain mode locked (FDML) laser operating at an axial scan (A-scan) rate of 1.68MHz, with an axial resolution of 9 μm (FWHM in tissue) and a lateral resolution of 20 μm ($\frac{1}{e^2}$ on the retina)^26^. For each patient, we scanned both eyes. The scan protocol included $n=2$ repetitions for every lateral scan (B-scan). The volumes consist of 1536 × 2048 × 2044 samples, corresponding to roughly 6 mm × 18 mm × 18 mm large scans on the retina. Each B-scan in the OCT volume was reconstructed and the OCTA data was generated from the complex variance of neighboring B-scans^26^. Generation of the OCTA en face image data is described in further detail in Supplementary (*OCTA data reconstruction*).

**Figure 1:**
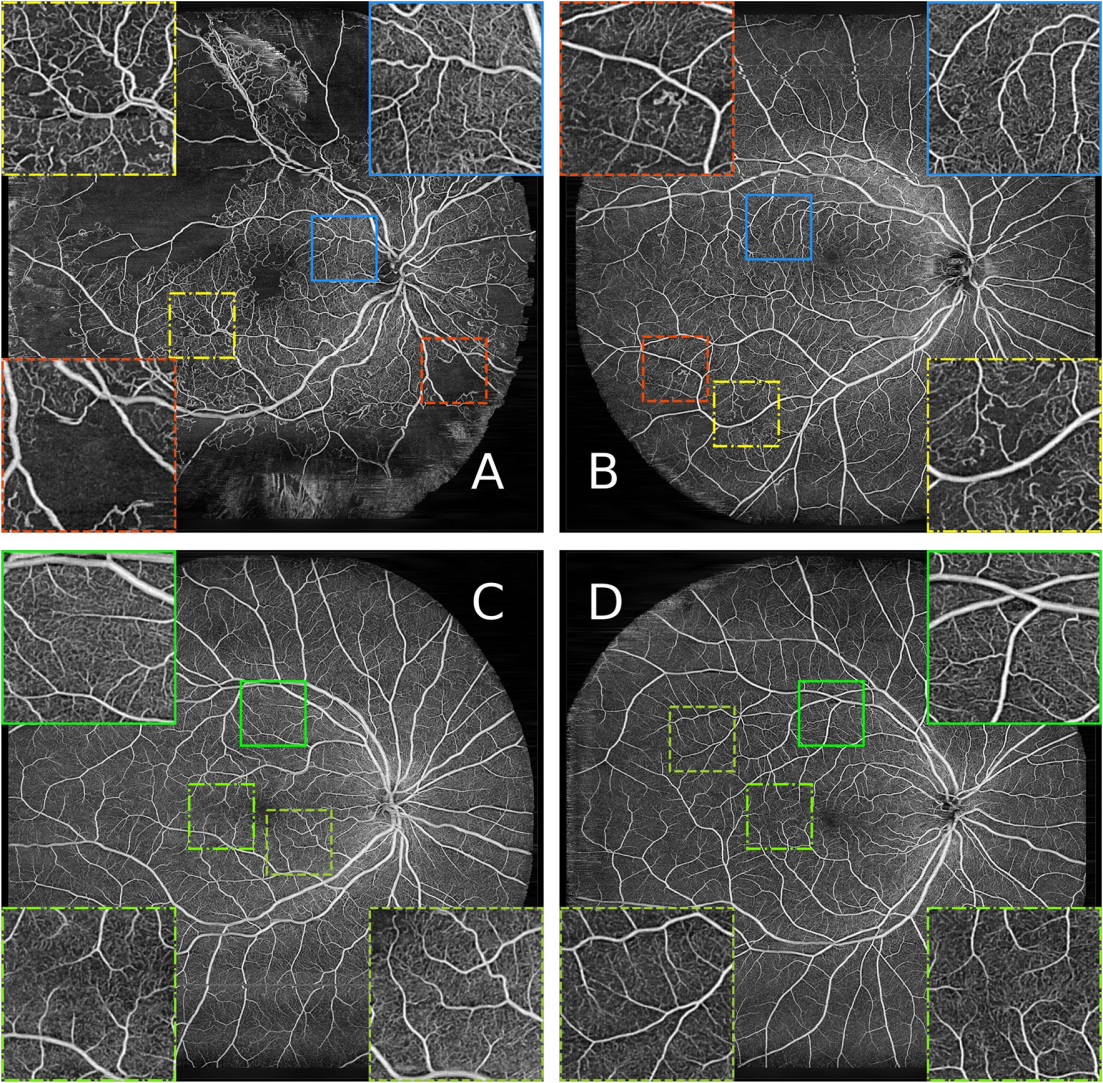
Typical OCTA en face images from the utilized dataset. Red (dashed) boxes denote exemplary ischemic areas, yellow (dashed-dotted) boxes highlight pathological vessels, blue (straight) boxes zoom-ins on areas with no obvious pathological changes to the vasculature, despite coming from an image showing clear indications of DR in other areas. The green boxes in (**C**) and (**D**) show areas that appear healthy, as neither of the scans shows obvious clinical markers, indicating DR. (**A**): shows an face image of a patient with severe signs of DR, (**B**) of a patient with mild sign of DR, (**C**) of a patient with DM but no signs of DR, and (**D**) from a healthy volunteer.

Healthy volunteers were also included in the clinical study which was cleared by the local ethics committee of the Medical University of Vienna. Participants were briefed by physicians and gave their informed consent for using their data for this work. The study was performed following the guidelines and regulations and in accordance with the declaration of Helsinki. The widefield OCTA scans were taken by trained personnel according to the protocol of the study setup to assure consistent quality of the data. For this study, we utilized en face images of 102 diabetic patients, most of which show clear clinical evidence of the presence of DR of some sort, i.e. biomarkers, and 40 healthy volunteers. In total, we used 352 OCTA en face images from diabetic patients and healthy volunteers. Some patients were imaged multiple times. The largest refractive error that was observed in this study was − 6 diopters, which could be imaged comfortably. Refractive errors could be corrected by operator adjustment of the optical system prior to imaging. We split the data into a training set (211 diabetic, 64 healthy), a validation set (24 and 24), and a test set (22 diabetic and 8 healthy). The test data set was kept as is for all stages and evaluation, to ensure comparability of the network’s predictive accuracy. The widefield OCTA en face images from our dataset include various kinds of appearances and stages of progression of DR and are illustrated in Fig. 1.

### Multiple instance learning with neural networks

We expect our data to hold information on an instance level. More precisely, smaller sub-patches, in MIL-terminology referred to as instances, of the OCTA en face images already contain most relevant DR-related biomarkers. Therefore, the general assumption is that, given an en face image (representing a bag in terms of MIL), a classifier should be able to detect the instances which carry the properties that contain all relevant information of their parent image. In instance-space MIL the bag is classified as an aggregation of individual instance ratings. This implies only local data is used to predict the global label, i.e. one can expect classification-relevant, discriminative features at the instance level. This criterion is often called meeting the standard multiple instance (SMI) assumption and means that instances of a positive class can only be found in positive bags. In our case, this means that we only expect to find instances carrying biomarkers of DR in diabetic-positive labeled bags. High instance-space training accuracies are, therefore, a clear sign of overfitting. On the other hand, high precision, i.e. close grouping of the predicted values, is desirable if the SMI assumption holds. This process, however, does not provide means for learning on a bag level. If again, the SMI assumption holds, bag classification can be carried out via the aggregation of instances, a process usually referred to as MIL-pooling. This rather simple bag-level classifier performs max-pooling on all instances of a bag: $p(X|y) = max(p(x_i|y))$, where $x_i$ are the feature spaces of a bag *X* and *y* their label. A way of making instance-space classification more robust is to include instances that potentially also hold valuable information, via e.g. the arithmetic means, or average pooling of the instance predictions. Instance-space classification is not effective if global information is important—this is information that all individual instances themselves would be lacking. Since this is not the case for our clinical dataset, we chose an approach that utilizes MIL-pooling. We show in chapter *Gaining intuition* in the Supplementary that our MIL-approach generalizes with a dataset with similar label noise distribution, for our proposed method, MIL-ResNet14.

#### Data pre-processing

**Figure 2:**
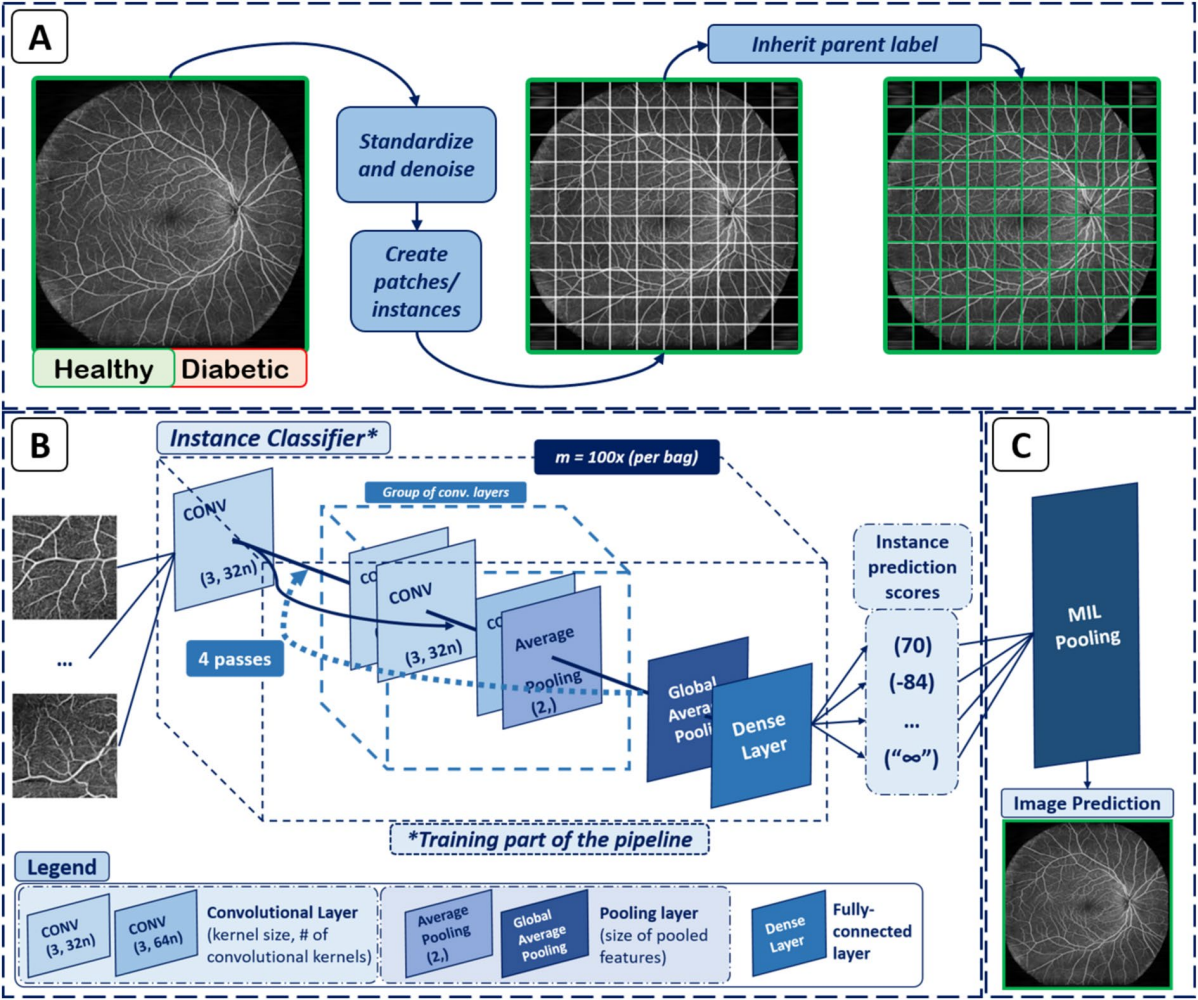
(**A**) Pre-processing of the OCTA en face images, i.e. bags. Every bag has a global label—whether or not the person is a diabetic. (**B**) Architecture of MIL-ResNet14 (MIL-ResNet14). All instances of an entire bag are passed into the networks’ first convolutional layer. The inner dashed cube indicates the subsequent four convolutional blocks, all identical in their design (dashed arrow hinting at the similar layout the data is passed through sequentially during training). (**C**) Post-processing, i.e. MIL-pooling—the average of the 3 highest instance scores is used to classify the entire bag.

In order to transform the OCTA en face dataset into a MIL context, we perform various pre-processing steps. In the first step all images are denoised (via a Gaussian filter with $\sigma =1$) and standardized, i.e. their gray values are centered such that the mean pixel value is zero and the standard deviation is one (see Fig. 2A). Every image is then divided into 100 $(10\times 10)$ sub-patches, the instances of the image (bag). This is done in a uniform checkerboard manner, with no regard for the local structure (see Fig. 2A). The resulting instances inherit the label of the full OCTA en face image. The MIL assumption implies that the resulting instance-level dataset exhibits asymmetric label noise and is very important for our MIL implementation. A positive class incorporates both, image-patches with and without DR-lesions. Meanwhile, the negative class consists only of instances that do not show any DR signs. Our entire software pipeline was implemented in *Python 3.10* and *TensorFlow 2.7.1*, and all training and inference operations were executed on an NVIDIA GeForce RTX 3080 Ti with 12 GB of memory.

#### MIL-ResNet14 model architecture and training

The MIL-ResNet14 model consists of a neural network (NN) that predicts status (meaning coming from a diabetic or non-diabetic person) of a single OCTA instance and a MIL-pooling model that agglomerates all instance predictions of one bag into a singular bag score. Its fundamental architecture (instance classifier) is based on ResNet14. Training is carried out on an instance basis. After being passed through an initial convolutional layer, each instance is passed through 4 consecutive blocks (indicated by the dashed box and arrow in Fig. 2), each consisting of three convolutional layers followed by average pooling. All convolutional layers have SeLU activation. Finally, an instance score is determined by global average pooling of each feature map followed by a dense layer (see Fig. 2B).

Binary cross entropy is used for loss calculation of MIL-ResNet14:

1 $$\begin{aligned} L_{MIL-ResNet14} = -\sum _i^n \sum _j^k Y_i \log ( {\hat{y}}_{ij} ) \end{aligned}$$

where *Y* denotes the bag label while ${\hat{y}}$ denotes the instance prediction score, *n* is the number of OCTA images and *k* the number of instances per bag. Several other publications on MIL in combination with NNs use MIL losses^43,44^. These perform MIL pooling prior to loss calculation and therefore resolve the label noise issue introduced by inheriting bag labels:

2 $$\begin{aligned} \begin{aligned} L_{pre-pool.} = -\sum _i^n Y_i \log ( M {\hat{y}}_{ij} ) = -\sum _i^n Y_i \log ( {\hat{Y}}_{i} ) \end{aligned} \end{aligned}$$

However, this reduces the positive effect of the increase in data size resulting from the instance generation. Therefore, in this work, the loss function is applied directly to the instance predictions. Consequently, if no overfitting occurs, accuracy is expected to be low, since it is negatively impacted by false label annotations.

Data augmentation during training is carried out in that instances are rotated (multiples of 90°), have their brightness and contrast adjusted, and are shuffled. Thus, training instances are assumed to be completely independent and no information about the bag affiliation is given to the network. The MIL pooling algorithm used is the mean of the three highest prediction scores, as this is a more robust max pooling operator. Because of the asymmetric label noise problem, class weights are calculated that prefer healthy instances by a factor of two per instance over diabetic instances. This helps to decrease the false positive rate while increasing the false negative rate, which however is acceptable and yields better bag-accuracy during training. The optimal threshold is determined by the maximum geometric mean of the receiver operating characteristic (ROC curve) of the validation dataset.

#### Benchmark architectures for comparison

We took two proven-capable network architectures, ResNet14 (identical to the instance-classifier portion we used in our approach) and VGG16, and trained them in a supervised (or SIL) manner to predict the classes of full en face OCTA images. VGG16 is initialized using ImageNet^45^ weights and only the last 6 layers are retrained. This approach has already yielded promising results in earlier work^46^. We trained both competing networks on the same data and performed hyperparameter tuning. For evaluation, we only took into account the versions of ResNet14 and VGG16 that generalized the best, i.e. performed the most accurately on classification on the test dataset. Training parameters varied between the different networks, especially between MIL-ResNet14 and ResNet14 & VGG16. ResNet14 consisted of 1,660,993 trainable parameters, VGG16 of 14,747,585 of which 7,112,321 were set to be trainable. Both networks’ input dimensions were 2044 × 2048, i.e the original image dimensions of the widefield OCTA en face images. MIL-ResNet14 consisted of 443,489 trainable parameters with input dimensions of 204 × 204.

The ROC evaluates the binary classification performance of a trained network with varying thresholds. The area under the curve (AUC) summarizes the ROC in one value. This metric is threshold-independent, which is important in case the two classes are not equally distributed. Label noise resulting from inheriting bag labels leads to instance-based performance metrics being less reliable. Therefore, during the validation step, MIL pooling is performed to obtain a noise-free bag metric. Since the instance dataset classes are also unbalanced and the actual number of samples per class is not known, the AUC is used as a bag metric: a metric to compare the networks’ “accuracy”. We do not merely use accuracy as a score, but instead, utilize ROC. ROC is used for binary classification problems, like predicting weather or not an OCTA en face image was taken from a diabetic patient or not. ROC plots the true positive rate over the false positive rate, often in a medical diagnostic context, referred to as the *probability of detection* against the *probability of false alarm*.

3 $$\begin{aligned} \begin{aligned} \text {AUC}_{MIL} = \text {AUC}(Y_i, \max ({\hat{y}}_j)_i) \end{aligned} \end{aligned}$$

with *Y* denoting the bag label, ${\hat{y}}$ an instance prediction and *i* and *j* bag and instance numbers respectively. The $\text {AUC}_{MIL}$ is used for early stopping, implying that the network weights which resulted in the largest $\text {AUC}_{MIL}$ are selected as the best network.

We describe the accuracy as the ratio of the number of correct predictions over the number of total predictions taking into account images from diabetic patients without signs of DR. We, therefore, refer to it as the “corrected” accuracy. “Corrected” precision denotes the percentage of correct predictions of images that actually display signs of DR. The F-score (F1-score) is calculated as the harmonic mean of precision and recall and we calculate it as $\frac{2 TP}{2 * TP + FP + FN}$, with TP being the *true positives*, FP *false positives*, and FN *false negatives*.

### Grad-CAM

Since we do not have any semantically annotated ground truth data available, we looked at the comparison of areas deemed important for classification by the networks. Which features a NN deems important is not easy to comprehend, especially with very deep CNNs. Understanding the underlying mechanisms leading to a particular classification can therefore be quite challenging for humans. Gradient-weighted class activation mapping (Grad-CAM) is a technique to visualize important regions for predictions in images and, ultimately, a CNNs decision-making process in a human understandable way^42^. It is capable of creating maps that highlight the spatial activity of feature maps. These images are often referred to as saliency maps or attention heat maps. A huge advantage of Grad-CAM is that it does not require the network to be retrained to generate these attention heatmaps^42^. More details about the generation of the heatmaps can be found in the Supplementary (*Gradient-weighted class activation mapping (Grad-CAM)*).

We overlaid the Grad-CAM attention heatmaps with the corresponding OCTA en face images to better understand which parts of the image the networks deemed important after inference. This way we can better assess if the network learned to pay attention to clinically meaningful areas. We also asked an ophthalmologist to label exemplary OCTA en face images for visual comparison with Grad-CAM overlay (see Supplementary*Widefield OCTA en face Grad-CAM overlays*).

ResNet14 and VGG16 are trained and perform inference of entire OCTA en face images, while our novel proposed network architecture, MIL-ResNet14, operates on instances, i.e. sub-patches. To compare both networks we stitched together all instances after they have been predicted by MIL-ResNet14.

### Adversarial attacks

CNN-based classifiers can be vulnerable to fluctuations in the input data^47^. This poses a potential problem for the classification of DR. Some techniques have proven to be particularly suitable for provoking misclassification, one of the more prominent ones being fast gradient signed method (FGSM)^48^. Contrast, absolute pixel values, and the distribution over the entire OCTA image can vary quite a lot depending on the exact settings during imaging and the instructions and alignment of the operator who took the scans. FGSM calculates the gradients of the network’s loss, with respect to the input image. Hereby we take a trained network’s weights $\Theta$, *x* the original input image, *y* the original input weights, *L*(...) represents the loss function and $\epsilon$ being a normalization factor, to ensure that the perturbations stay minimal:

$$\begin{aligned} I_{adv,FGSD} = x + \varepsilon * sign(\nabla _{x} L(\Theta ,x,y,)) \end{aligned}$$

We deem this method especially interesting for testing a network’s robustness since it operates on the weights of the respective prediction of an image and is applied to the trained network. After artificially increasing the ‘classification noise’ of the test data with $\epsilon =0.1$ the accuracy of the classification is re-evaluated. We evaluate this expected drop in accuracy for our network, MIL-ResNet14, and compare it to ResNet14.

## Results

In this chapter, we show the results of the individual performances and comparison of all three NNs we trained and evaluated, ResNet14, VGG16, and MIL-ResNet14. All networks were tuned to achieve the highest classification performance in terms of numerical scores. Additionally, we evaluated the details which contributed the most to the decision process in classifying the widefield OCTA en face images, via Grad-CAM overlays. Thus we could review which areas in the images were crucial for the classification process and if they incorporate relevant lesions. Lastly, since the quality of clinical OCTA en face images can fluctuate quite considerably, we evaluated the robustness of MIL and ResNet14 against artificially introduced noise to the test dataset via adversarial attacks, namely FGSM.

### MIL-ResNet14 performance

**Figure 3:**
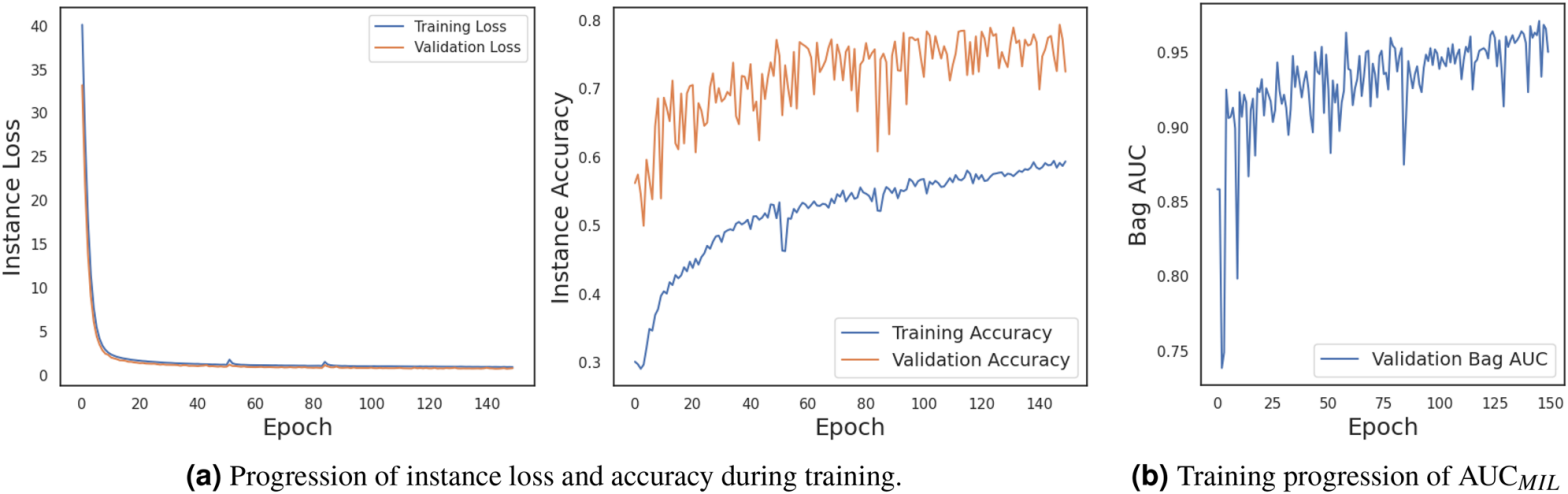
Training progression for instances and bags of MIL-ResNet14 on the clinical data set. MIL-ResNet14 was trained until Bag AUC stopped improving.

**Figure 4:**
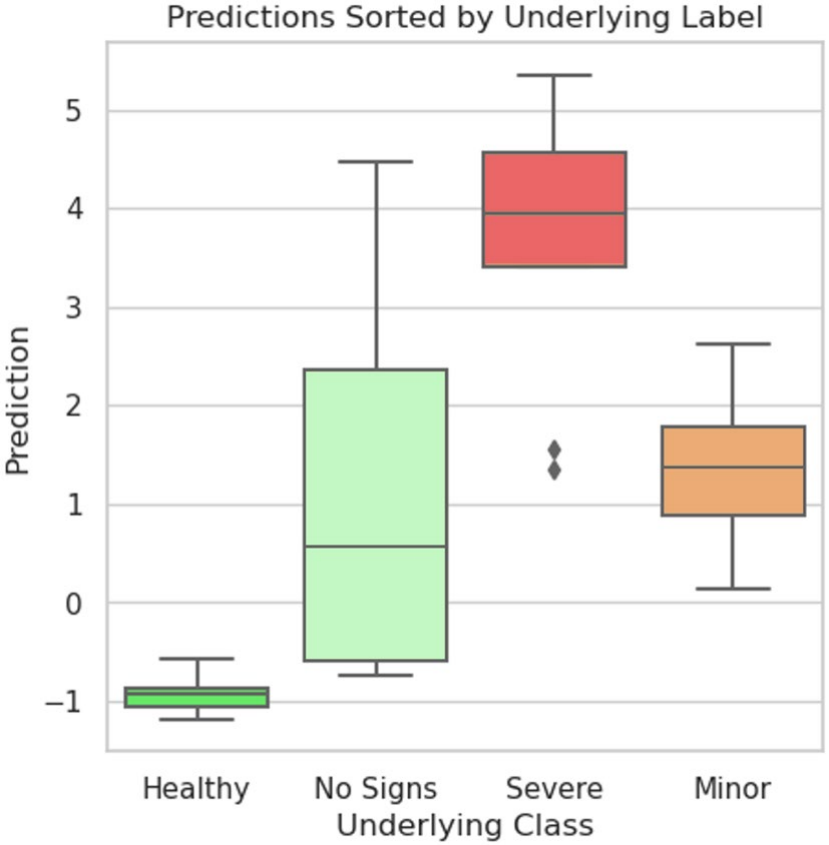
Bag prediction scores of the test dataset, sorted after their underlying label. Boxplots indicate the distribution of the individual instances coming from their respective classes. High prediction scores indicate a high probability that the image contains signs of DR. Bags containing instances with very strong signs of DR are classified higher than bags with only minor DR signs.

In this section, it is evaluated if our instance-space MIL approach is suitable for automatic detection of DR in OCTA en face images. Figure 3a shows the training progression of the model. The instance accuracy is low due to label noise on an instance level. Notably, the validation accuracy is higher only because the validation dataset consists of proportionally more images labeled as healthy, which exhibit less label noise. Network weights for evaluation are selected according to the epoch with the highest bag AUC (see Fig. 3). On a bag level, instance label noise does not exist, which makes AUC a good indicator to avoid overfitting on the training dataset. The highest $\text {AUC}_{MIL}$ was achieved after 8 epochs. The test accuracy on an instance level is 68.8% and after MIL pooling the bag AUC reaches 0.989. After the optimal threshold is calculated, the test accuracy reaches 0.967, with the one falsely classified diabetic OCTA image showing no visible DR signs (Fig. 4).

**Figure 5:**
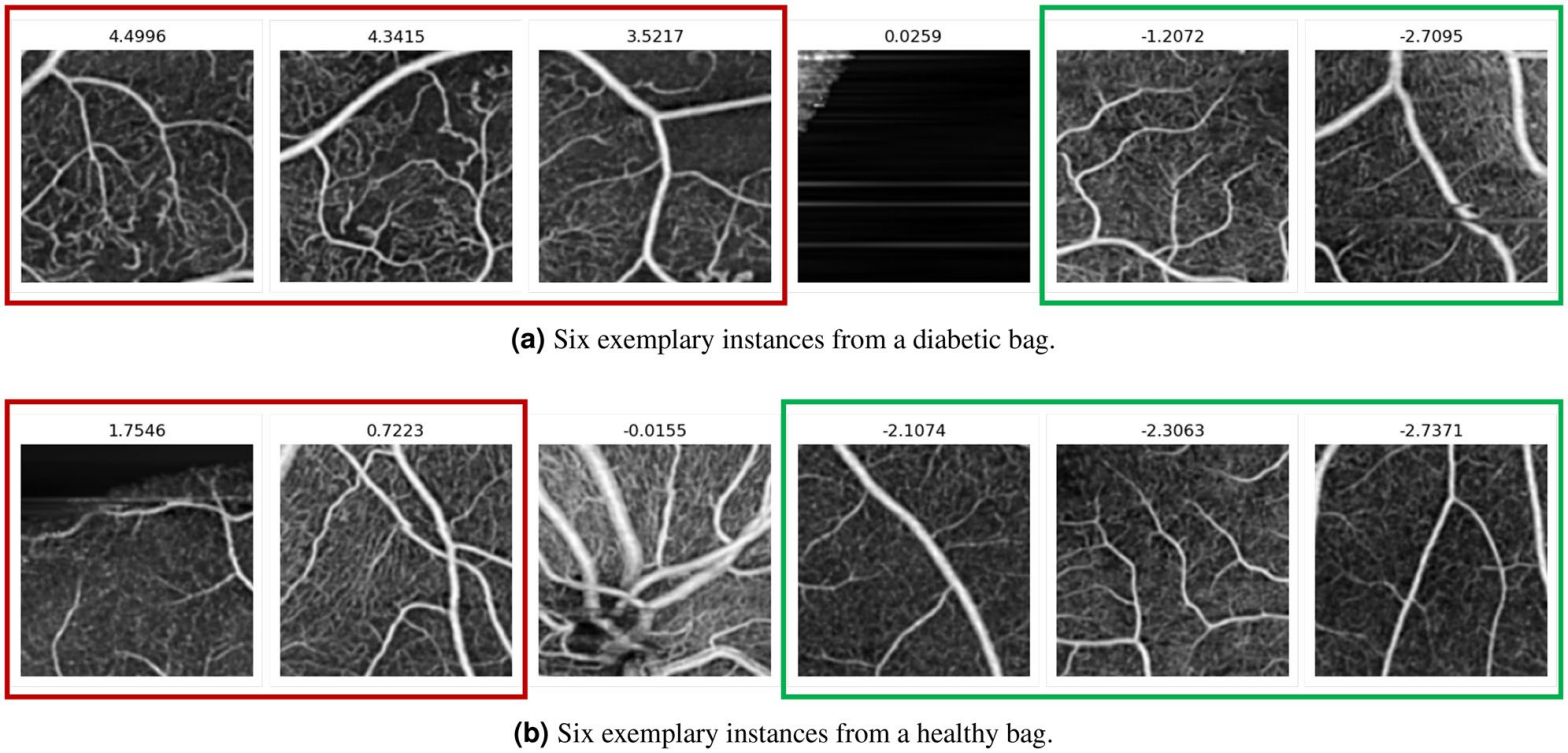
Prediction scores of MIL-ResNet14. Red bounding boxes mark the instances of one bag with the highest prediction scores (probably diabetic), green boxes the instances with the lowest scores (probably healthy), and scores close to zero indicate instances that occur in both cases.

The classification performance of MIL-ResNet14 is evaluated on 30 OCTA en face images that were not used in training. Figure 5 shows instance prediction scores of two exemplary test images, one positive and one negative. The network correctly identifies instances with distinct DR lesions (see Fig. 5a) by assigning to them the largest prediction scores of the bag. Additionally, absolute values of positively classified instances are notably smaller (0.72–1.76) in a negative, i.e. healthy classified bag (Fig. 5b), than positive instances in a positively, i.e. as diabetic, classified (3.52–4.5) bag (Fig. 5a). Instances that were classified as healthy, on the other hand, were classified with low (negative) prediction scores in both bags. Additionally, instances that occur with the same probability in the diabetic and healthy class, like background or the optic nerve head (ONH) (both without red or green bounding boxes in Fig. 5), were predicted as neither diabetic nor healthy, with values close to 0.

**Figure 6:**
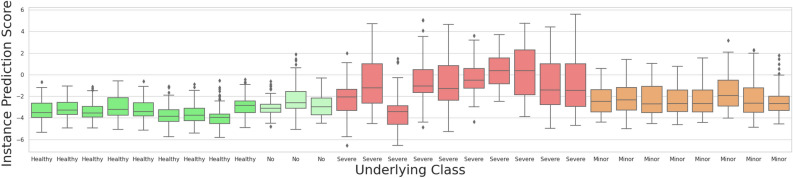
Distribution of predicted instances in the tested dataset. Coloring represents the degree of DR severity of each bag. Green for healthy bags, light green for diabetics without DR, orange for mild DR, and red for severe. Each box plot shows all of the 100 instance prediction scores for an entire bag.

Our test dataset consists of 30 images in total, 22 of those from diabetic patients. This set shows an ideal distribution of appearances of the various classes healthy, diabetic but no signs of DR, minor signs of DR, and severe signs of DR (see Fig. 1). The exact distribution of all prediction values for all individual instances are shown as boxplots in Fig. 6. As can be seen, all bag feature instances that are predicted by MIL-ResNet14 as healthy. In the severe class, these stem from background instances or locally healthy tissue. Figure 6 also shows that the median prediction value of the minor class is very close to the median of the healthy class. This is reasonable since early DR cases are determined by only a few, local abnormal areas which in turn can be seen in the higher maximum instance prediction of the minor class. When evaluating this data, one should keep in mind that the four subjective categories we chose for comparison have smooth transitions in terms of their biomarkers.

Figure 4 displays a summary of the bag predictions of MIL-ResNet14 for our test dataset, which we divided up into the aforementioned four categories for signs of DR. The groups shown in Fig. 4 were tested for distinctness using a Kruskal-Wallis test (non-parametric ANOVA). The results of the test showed that the groups were sufficiently different from each other (p-value < 0.0001). It is worth noting, however, that the total sample size of 30 images and the unequal distribution of the samples per group should not lead one to over-interpret the significance of this result. After MIL-pooling the severe class clearly shows the largest prediction scores. Meanwhile, the no signs class exhibits a median value well below zero and close to the healthy class. All scans stemming from healthy volunteers show prediction scores below 0 and MIL-ResNet14 is, therefore, capable of confidently identifying them.

### Comparison with other architectures

Since we wanted to compare our approach to networks that proved to achieve very high classification accuracy in the past, we evaluated all three models based on their “corrected” accuracy, “corrected” precision, F-score, and their ROC-AUC scores. The hyperparameters of all networks were tuned to achieve the highest ROC-AUC scores individually. Numerical scores on our utilized clinical widefield OCTA test dataset are shown in Table 1.

**Table 1:** Numerical values of the different networks architectures compared with each other.

Validation metric	VGG16	ResNet14	MIL-ResNet14
F1-score	0.857	0.909	0.950
ROC-AUC	1.0	0.942	0.973
“Corrected” (bag) accuracy	0.6	0.733	0.9
“Corrected” (bag) precision	0.555	0.666	0.909
Instance accuracy	–	–	0.541
Instance precision	–	–	0.993

Instance-level metrics were only possible to determine for MIL-ResNet14. The hyperparameters of all networks were fine-tuned and the displayed scores are derived from the networks with the best performance on the test dataset.

Notably, MIL-ResNet14 performs the best out of all three compared architectures for the bag-level scores, specifically for the “corrected” precision, “corrected” accuracy, and the F-score. The scores for both, “corrected” accuracy and “corrected” precision correct for the classification of images from diabetic patients which do not display any signs of DR. MIL-ResNet14 reached an instance accuracy of 0.541 and an instance precision score of 0.993. VGG16 displayed a ROC-AUC of 1.0, which indicates that positive and negative predictions are perfectly separable. VGG16’s classification accuracy was however worse than the ones of ResNet14 and MIL-ResNet14.

After being subject of adversarial attacks the “corrected” accuracy of ResNet14 dropped to 0.528, while MIL-ResNet14’s “corrected” (bag) accuracy dropped to 0.9. Most of the same highly ranked instances kept their meaning for the bag classification.

#### Visual agreement with Grad-CAM heatmaps

**Figure 7:**
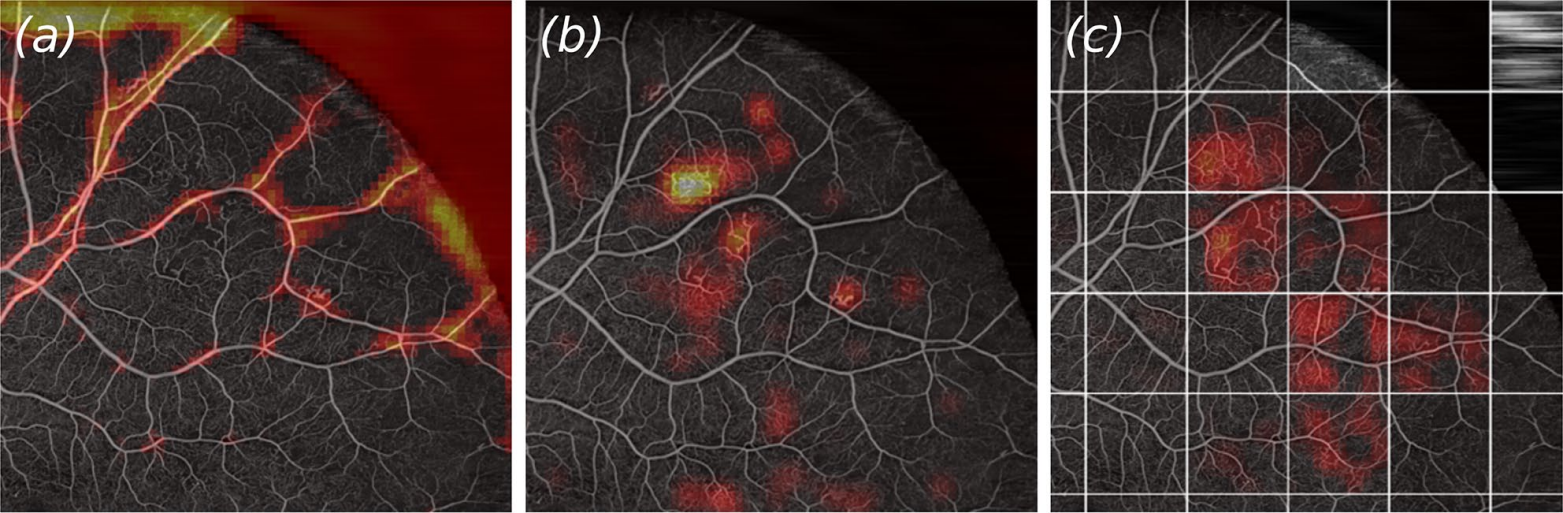
Example patch of an eye displaying weak signs of DR. Overlays of Grad-CAM saliency maps onto (**a**) ResNet14, (**b**) VGG16, and (**c**) MIL-ResNet14. Instances on which MIL-ResNet14 performed inference in (**c**) were stitched together to display the same global context for all three network architectures.

It is notoriously difficult to obtain precise annotated clinical data, especially for large images like the utilized widefield OCTA dataset. These highly detailed, remarkably large datasets are particularly time-consuming and cumbersome to annotate. While the SIL-based networks, ResNet14 and VGG16, achieved somewhat similar AUC scores, the image details highlighted by Grad-CAM which played a large role in the decision making-process differed between all three architectures. Figure 7 shows the comparison for an example OCTA-patch exhibiting mild signs of DR. ResNet14 (Fig. 7a) had a high feature activity in the periphery on large, presumably healthy vessels, as well as the background. VGG16 on the contrary (Fig. 7b) seemed to focus on areas displaying abnormal vascular structures, especially clogged vessels, which is a proven biomarker for DR in OCTA en face images. MIL-ResNet14’s Grad-CAM overlays, displayed in the right-hand-side panel (c) of Fig. 7, seemed to pay attention to abnormal vascular structures, as well as ischemic areas, the two most important biomarkers visible in the patch displayed in Fig. 7.

**Figure 8:**
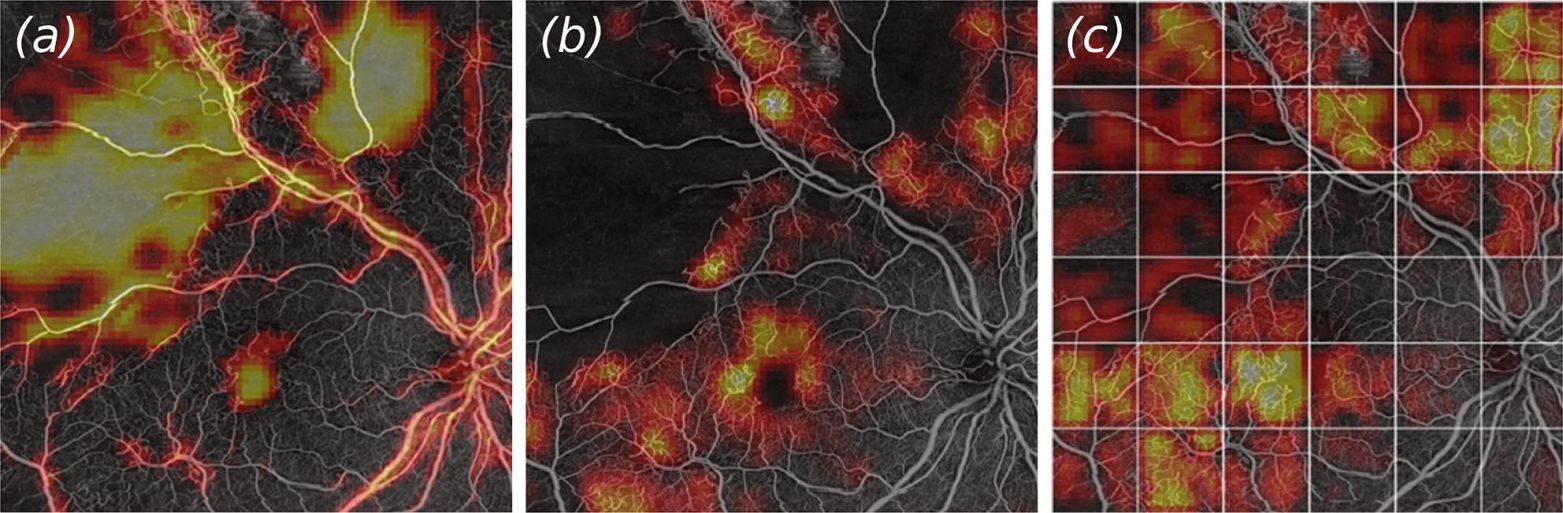
Example patch of an eye displaying severe signs of DR. Overlays of Grad-CAM saliency maps onto (**a**) ResNet14, (**b**) VGG16, and (**c**) MIL-ResNet14. Instances on which MIL-ResNet14 performed inference in (**c**) were stitched together to display the same global context for all three network architectures.

For comparison of the Grad-CAM overlays in different cases, we looked at the heatmaps of another typical OCTA image from our test dataset, displaying clear signs of severe DR. ResNet14, displayed in the left-hand-side panel (a) of Fig. 8 had a high feature activity score in the large ischemic areas, the fovea, and around the large vessels of the ONH. VGG16 (Fig. 8b) seemed again having focused on abnormal vascular structures, while seemingly ignoring the large ischemic areas. MIL-ResNet14 considered instances with both, ischemic features and vascular areas of abnormal appearance highly relevant in terms of feature values within the Grad-CAM heatmaps.

It could be observed as a general trend that ResNet14, even though achieving reasonably high classification accuracy, seems to pay attention to features in the scans that are either not showing relevant features, or solely paying attention towards large ischemic areas. VGG16 on the other hand seems to decide mostly based on abnormal vasculature, while ignoring ischemic areas. MIL-ResNet14 seems to pick up on both biomarkers, ischemic areas, as well as anomalous fine-vasculature, while ignoring areas which so not hold relevant information for classification.

In order to gain further insight into the decision-making process of the networks, we asked an experienced clinician to annotate three OCTA en face images in terms of three visible biomarkers, vascular anomalies/dropouts, ischemic areas, and microaneurysms/neovascularization. To visually compare the areas the evaluated networks paid attention to, we again overlaid the Grad-CAM saliency maps onto the entire annotated images, which can be seen in the Supplementary (*Widefield OCTA en face Grad-CAM overlays*). The general trends in patterns of Grad-CAM for all three networks discussed in this section can also be observed in this case, although to a lesser extent.

## Discussion

In this manuscript, we presented an approach for the classification of DR features in a weakly-labeled clinical widefield OCTA en face retina dataset, utilizing a MIL-based classification algorithm. We could show that our CNN-based MIL classification network, MIL-ResNet14, is capable of detecting relevant DR biomarkers. We verified this by comparing the classification accuracy to proven-capable, state-of-the-art networks for this task and Grad-CAM-generated visual heatmap overlays onto the en face images. We could show that, with our approach, the need for cumbersome and labor-intensive precise annotations can be circumvented. MIL-ResNet14 achieved superior classification scores in terms of F1-score, with a high ROC-AUC score, and pays more thorough attention to clinically relevant features in the images.

MIL-ResNet14 outperformed both other state-of-the-art SIL-based classifiers we trained and optimized for comparison. MIL-ResNet14 reached a ROC-AUC of 0.974, compared to ResNet14’s 0.905 and VGG16’s 1.0. Even though VGG16 achieved an AUC-perfect score, its classification metrics were inferior to MIL-ResNet14’s. High values for precision, and relatively poor values for instance-accuracy show good agreement with the SMI assumption and serve as a good indicator that MIL-ResNet14 was not subject to overfitting. MIL-ResNet14 maintained a “corrected” accuracy of 0.9 after being subject to adversarial attacks. For ResNet14, on the other hand, “corrected” accuracy dropped to 0.528. We, therefore, deduce that variations in a clinical dataset due to e.g., inaccurate patient alignment do not affect MIL-ResNet14s ability to accurately detect scans from diabetic patients.

Identifying clinically relevant lesions and labeling these in OCTA en face images requires clinical expert knowledge of the appearance of various markers like neovascularization, microaneurysms, enlargement of the foveal avascular zone, as well as ischemic areas. Figures 7 and 8 show that MIL-ResNet14 performs well in terms of picking up on these relevant biomarkers while ignoring areas that do not hold relevant information for the process. ResNet14 on the contrary picked up very well on ischemic areas, but also considered healthy vessels important. Regardless of the actual meaning of the heatmap values, it does not make any sense that a network pays attention to the background or healthy vessels close to the ONH. VGG16 seems to completely ignore both structures, to which ResNet14 paid close attention. Ischemic areas might not get recognized by VGG16 due to the fact that it was pre-trained on ImageNet, where purely black areas might not make sense as features. VGG16, however, was able to detect abnormal-looking vasculature accurately. Grad-CAM, with its heatmaps, provides humans with nicely interpretable visuals but the meaning of the saliency maps should not be over-interpreted for clinical decision-making.

While we do believe that information about which semantic areas in the scans were deemed important by the classifier is a good complimentary tool, the magnitude and underlying significance of these areas might not be easily comprehended.

We could demonstrate that our approach generalizes well for datasets exhibiting similar label noise as our clinical one (see Supplementary*Gaining intuition*). This represents a strong indication that our proposed MIL-approach is a reliable method for identifying pathologic changes in the retina via widefield OCTA en face images. The smaller receptive field of MIL-ResNet14 also might contribute to the fact that it was more capable of detecting lesions, compared to ResNet14 and VGG16.

As with most datasets, there are a couple of limitations within our utilized clinical widefield OCTA en face dataset that should be discussed. The en face images represent a projection along the optical z-axis of the OCTA volumes along a segmented boundary layer. During segmentation, some clinical markers get lost. This fact affects the performance of all networks. However, it affects all equally, since all are trained on the same data. Artifacts or pathologic changes in the OCTA volumes may lead to inaccurate segmentation and consequentially to artifacts in the en face images. Segmentation errors in the OCTA volumes are the major source of error or bias when solely using OCTA en face images. This is especially apparent when it comes to evaluating ischemic areas. Inaccurate segmentation can lead to “false ischemic” areas, meaning areas where vasculature is still present, but not displayed in the en face images. This leads to the display of a ‘false positive’ biomarker. The same is true for vascular anomalies and especially neovascularizations. The level of subjectivity in terms of providing an accurate annotation is therefore very high. However, since we do not rely on accurate ground truth data, but instead use weak bag-labels only, this does not pose a limitation of our presented method.

MIL-ResNet14 poses a reliable architecture to classify OCTA en face images with high intrinsic label noise. However, when using this approach one has to be careful with the training of the network. Common metrics during training, like the instance accuracy, for MIL-ResNet14 0.541, might give false readings if one is unaware that the comparatively low scores are indicative that the MIL assumption holds. Applying and comparing a MIL classification approach to a multi-modal image dataset consisting of OCT/OCTA and FP images would also be of high research interest.

Colored images of the patients used in this study were not available. The comparison of these images or even the incorporation of them into our MIL approach is something that we plan to look into for the continuation of this work. OCTA allows to visualize small capillaries and therefore allows to detect DR at an earlier stage. However, for more reliable detection of the lesions, the whole 3D data set should be utilized.

Due to not possessing ground truth labeled data of all lesions, we did not quantify whether or not MIL-ResNet14 is capable of highlighting all areas indicating them. The saliency map overlays generated by Grad-CAM could still serve as a rough tool for the localization of anomalies, to help guide a screener’s attention to areas of clinical interest in OCTA en face images. We conclude that our proposed method, MIL-ResNet14, serves as a reliable pre-screening tool for DR, to help untrained personnel to make decisions on the treatment or referral of early-stage DR, and can also serve as a tool to monitor the progression of the disease longitudinally.

## Supplementary Information

Supplementary Information.

## Data Availability

All data we utilized to train and evaluate the networks were taken from a clinical study, and we can therefore not provide the data publicly. Since OCTA retina data contain biometric information they fall under the GDPR.

## Code availability

The code base^49^, including all pre- and post-processors, test scripts (Jupyter Notebooks), and networks, is publicly available on Github https://github.com/LilWeirdProgramms/oct-classifier.
