# Supplementary material for: Multiple instance learning based classification of diabetic retinopathy in weakly-labeled widefield OCTA en face images
Source: Sci Rep. 2023 May 29;13:8713. doi: 10.1038/s41598-023-35713-4 (PMC10226980; doi:10.1038/s41598-023-35713-4)
Supplement: Supplementary file 1 — Supplementary Information. [file 41598_2023_35713_MOESM1_ESM.pdf]

Supplementary material for

Multiple instance learning based classification of diabetic retinopathy in weakly-labeled widefield OCTA en face images

Philipp Matten[1,‡,*], Julius Scherer[1,‡], Thomas Schlegl[1], Jonas Nienhaus[1], Heiko Stino[2], Michael Niederleithner[1], Ursula M. Schmidt-Erfurth[2], Rainer A. Leitgeb[1], Wolfgang Drexler[1], Andreas Pollreisz[2], and Tilman Schmoll[1,3]

[1]Center for Medical Physics and Biomedical Engineering, Medical University of Vienna, Waehringer Guertel 18-20 (4L), 1090 Vienna, Austria
[2]Department of Ophthalmology and Optometry, Medical University of Vienna, Waehringer Guertel 18-20, 1090 Vienna, Austria
[3]Carl Zeiss Meditec Inc, 5300 Central Pkwy, Dublin, CA 94568, USA
[*]philipp.matten@meduniwien.ac.at
[‡]These authors contributed equally to this work

OCTA data reconstruction

In the pipeline we utilized, widefield optical coherence tomography angiography (Widefield OCTA) data are reconstructed as the variance of n pairs of line-scanned tomograms, subsequently acquired in stacks. These line-scanned tomograms, so called B-scans, consist of multiple, adjacent scans in optical z-direction (so called A-scans) and are recorded at the same physical location. These are referred to as B-scan repetitions. For every voxel v in every sample in a stack of B-scan repetitions, i.e. a sequence of n repetitions, before incrementing the scanning to the next location and recording again a sequence of n repetitions, we calculate the flow signal of consecutive B-scans as follows:

$$f_{x,y,z} = log_{10}\left(\frac{1}{n-1}\sum_{i=1}^{n-1}|v_{x,y,z,i} - v_{x,y,z,(i+1)}|^2\right) \tag{1}$$

Each voxel $v_{x,y,z,i}$ and $v_{x,y,z,(i+1)}$ is registered and corrected for phase-shifts (A-scan-wise $z+\delta z$), before (1) is applied. n is the number of B-scan repetitions to calculate the flow from and in our case, n always defaults to 2. All voxels $f_{x,y,z}$ in the resulting flow cube represent the registered optical coherence tomography angiography (OCTA) of a complete volume of one eye of a patient or volunteer.

After reconstruction of the OCTA volume scans the optic nerve head (ONH) is segmented via a U-Net-inspired neural network (NN) and excluded for the segmentation, as its composition impairs segmentation. A graph-cut based algorithm (modified Dijkstra's algorithm) segments the retinal pigment epithelium (RPE), and flattens the scan along the RPE and internal limiting membrane (ILM), twice each. The projection en face maps are then generated as the maximum intensity projection form the ILM to the photoreceptor inner and outer segment junction. These en face maps or images are then saved containing a randomly generated number (anonymized scan identification), and whether or not they come from a patient suffering from Diabetes Mellitus (DM) or a healthy volunteer.

Gradient-weighted class activation mapping (Grad-CAM)

Gradient-weighted class activation mapping is a technique which can be used to visualize the areas a convolutional neural network (CNN)-based classifier deems important for the class-decision process. Grad-CAM is therefore capable of producing so called *saliency maps* or *heatmaps*, which are generated by calculating the class-discriminative gradients after the last convolutional layer of a CNN. Outputs after fully-connected layers are essential for a classifier to make a decision. However, after this step spatial affiliation of the feature space is lost. Therefore one has to utilize Grad-CAM before the final prediction layer. Grad-CAM has been described as $L^c_{Grad-CAM}$, which is the class-discriminative localization map:

$$L^c_{Grad-CAM} = ReLU\left(\sum_k \alpha^c_k A^k\right), with \quad \alpha^c_k = \frac{1}{Z}\sum_i \sum_j \frac{\delta y^c}{\delta A^k_{ij}} \tag{2}$$

here α^c_k describes the average-pooled gradients of the network after backpropagation, with $\frac{\delta y^c}{\delta y^k_{ij}}$ being the feature activation map, A^k_{ij} the activation map at a specific location (i, j) (width and height dimensions), for a specific class c. For a specific attention heatmap of a certain class c one is only interested in those features that have a positive influence on the class-decision making, and the product of $\alpha^c_k A^k$ is therefore passed through *ReLU* activation. Examples of heatmap overlays from OCTA en face images can be seen in Figure 4 **(d)-(l)**. The resolution of the attention heatmaps differs between ResNet14 & VGG16, which perform inference on the entire OCTA en face image, and MIL-ResNet14. This is due to the fact that feature space dimensions between the two approaches are different, since image input sizes also differ. To compensate for that, we interpolate both heatmaps for a more comparable visual overlay (see Figure 4).

Gaining intuition

Multiple instance learning (MIL) for classification of weakly-labeled medical imaging data has, to the best of our knowledge, not been studied too extensively. We therefore want to show in the following section that this approach of accurately predicting image (bag) labels from noisy sub-patches (instances) actually generalizes very well, assuming that the MIL assumption holds. By dividing images into sub-patches and inheriting the parent label with no regards to the actual patch content, label noise is created on an instance level. Figure 1b shows a sub-patch, that is labeled diabetic, even though it exclusively shows background. Similarly, Figure 1c shows healthy vasculature, but is labeled diabetic since it stems from a bag with diabetic retinopathy (DR). Figure 1d, in contrast, also shows a healthy patch, and is labeled as healthy, as it comes from a bag of a healthy volunteer. This discrepancy of the actual instance label and the *ground truth* is referred to as label noise (see Figure 1b and 1c). In order to understand how a CNN-based classifier responds to this kind of noise, in a first experiment, a similar dataset is constructed from the MNIST image dataset. We evaluated this approach on our MIL-ResNet14 (MIL-ResNet14) architecture.

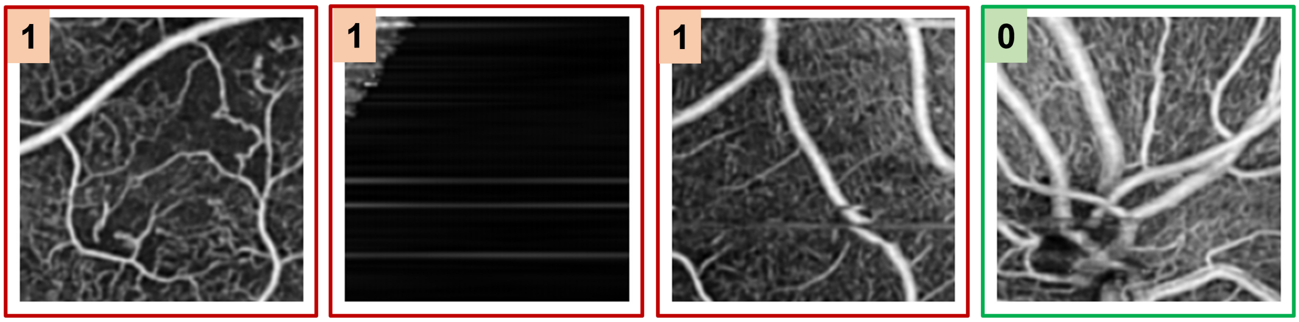

(a) DR signs found and labeled diabetic **(b)** Background, but labeled diabetic **(c)** No DR signs but labeled diabetic **(d)** No DR signs and labeled healthy

Supplementary Figure 1. Examples of instance-space label noise (1b, 1c) found in the derived dataset.

To show that this distribution of intrinsic label noise actually does not pose a problem for MIL to still learn to classifiy accurately, we present a MIL-MNIST dataset with the help of which we could show that our proposed method actually works in a more generalized setting. The MNIST dataset is a collection of digitized, handwritten numbers that are used to train and test machine learning (ML) algorithms, often number recognition systems. Our dataset is constructed by placing each MNIST number as an instance in a bag of size five MNIST numbers. A bag is labeled positive if it contains at least one image labeled '5' and negative otherwise. Instances inherit their bag label and are fed randomly into a three layer CNN. An exemplary result is presented in Figure 2. Due to bagging, several MNIST instances are, on an instance level, labeled false positive. For example, the MNIST number '6' is sometimes in the positive class and sometimes in the negative class, depending on the content of the remaining bag. However, the only instance with a high prediction score is MNIST number '5', which is the instance that determines the bag label. Figure 3b shows a box plot distribution of all instance scores. Images originally labeled '5' are predicted significantly higher than other images, which results in an accuracy of 0.9712 and an area under the curve (AUC) of 0.9862. This result indicates that the underlying instance concept is learnable by solely relying on bag labels. To visualize the

influence of bagging and label inheritance, the result of a binary MNIST dataset is shown in Figure 3a. In this dataset, an image is labeled positive if it is a '5' and negative otherwise. It can be seen that bagging and the accompanying label noise do not decrease the network's performance to detect images labeled '5'. It rather shifts all other prediction scores towards zero, since those predictions are less reliable.

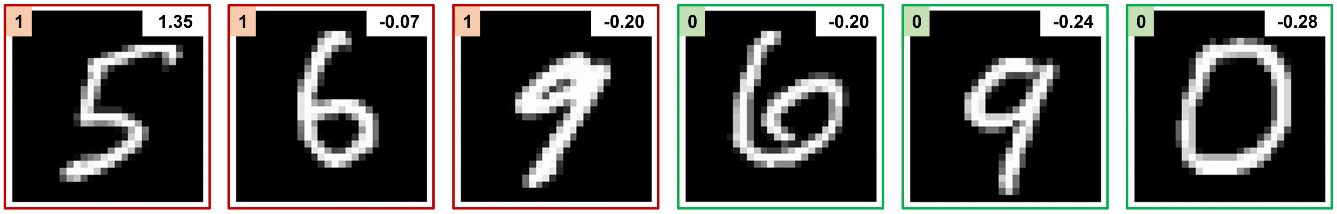

Supplementary Figure 2. Exemplary MIL-MNIST results with the instance label in the top left and the instance prediction in the top right. '5' is the underlying instance concept.

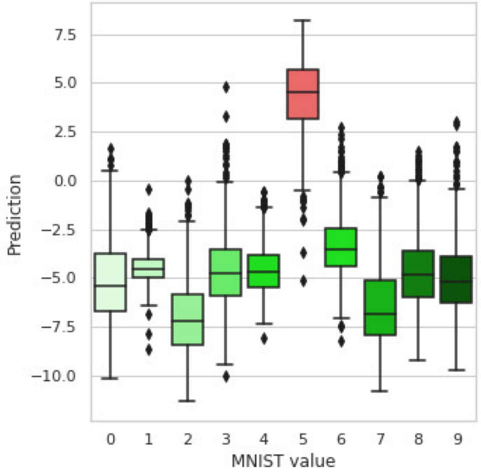

(a) Binary-MNIST dataset, classifying '5' against all other numbers.

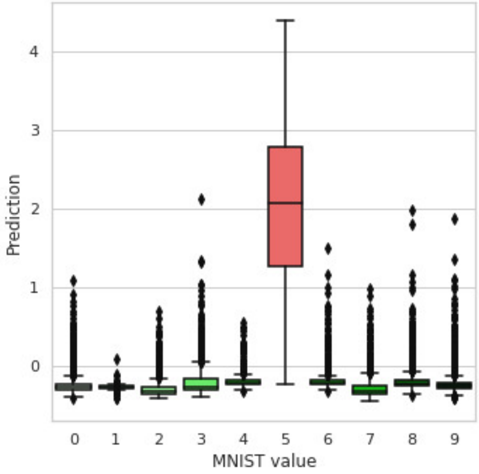

(b) MIL-MNIST dataset showing the influence of label noise through bagging.

Supplementary Figure 3. Box plot of prediction scores sorted by MNIST image label. A high prediction score indicates a possible '5'.

However, label noise also has a significant drawback. Since the underlying concept is learned, but the annotations are not reliable, metrics are not reliable as well. This results in a low reported instance accuracy of 0.6812, which is only increased by having knowledge about the instance level annotations. Therefore, we assume, one-sided label noise is rather an issue regarding model assessment, than it is regarding classification performance. We could show that the intrinsic label noise that is present in our dataset suffices the Standard Multiple Instance (SMI) assumption. Based on an MNIST dataset we could show that our MIL-based method generalizes very well, even for a different binary classification task.

Widefield OCTA en face Grad-CAM overlays

For the sake of providing thorough means of discussion - also of the limitations - of the information content of Grad-CAM, we show the entire Widefield OCTA overlays of three annotated en face images and the respective Grad-CAM heatmap overlays after all three networks we trained and evaluated performed inference on these images. All three images were part of our test dataset and omitted from the training of all three evaluated networks. Annotations in the images were provided by an experienced OCTA grader and ophthalmologist from the Department of Ophthalmology and Optometry at the Medical University of Vienna. Annotated images in Figure 4 show three cases of DR: **(a)**: severe (Pat.#1), **(b)**: mild signs (Pat.#2) and **(c)**: mild signs (Pat.#3). Red circles highlight annotated aneurysms, orange-marked and highlighted vessels show clogged capillaries, and blue areas show and highlight ischemic areas in the annotated images **(a)**-**(c)** in Figure 4.

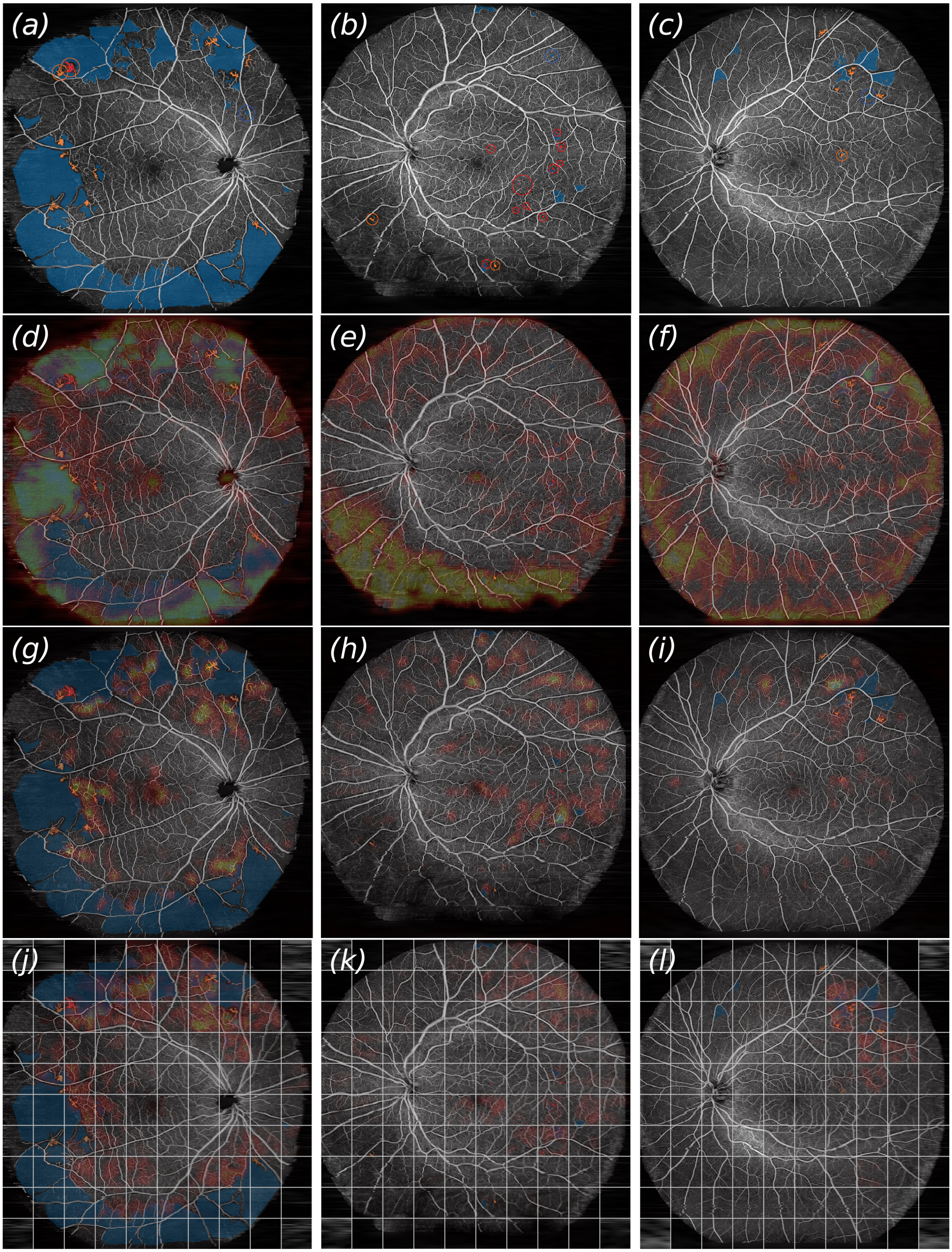

Supplementary Figure 4. Overlay of annotate clinical scans with the respective Grad-CAM saliency maps for the three different evaluated CNN-based classifiers. The left-hand-side column ((**a**),(**d**),(**g**), and (**j**)) are the respective overlays of Pat.#1 (severe signs of DR), the middle column ((**b**),(**e**),(**h**), and (**k**)) of Pat.#2 (mild signs of DR), and the right-hand-side column ((**c**),(**f**),(**i**), and (**l**)) of Pat.#3 (mild signs of DR). Row (**d**)-(**f**) are the Grad-CAM overlays for ResNet14, (**g**)-(**i**) of VGG16, (**j**)-(**l**) of MIL-ResNet14. Blue areas are ischemic, orange areas are occluded vessels, and red areas are aneurysms.
